# Production of biliverdin by biotransformation of exogenous heme using recombinant *Pichia pastoris* cells

**DOI:** 10.1186/s40643-024-00736-w

**Published:** 2024-02-01

**Authors:** Jianfeng Mei, Yanchao Han, Shihang Zhuang, Zhikai Yang, Yu Yi, Guoqing Ying

**Affiliations:** College of Pharmaceutical Science, Zhejiang University of Technology, 18 Chaowang Road, Gongshu District, Hangzhou, 310014 Zhejiang China

**Keywords:** Biliverdin, Biotransformation, Heme, Heme oxygenase-1, Recombinant *Pichia pastoris*

## Abstract

Biliverdin, a bile pigment hydrolyzed from heme by heme oxygenase (HO), serves multiple functions in the human body, including antioxidant, anti-inflammatory, and immune response inhibitory activities. Biliverdin has great potential as a clinical drug; however, no economic and efficient production method is available currently. Therefore, the production of biliverdin by the biotransformation of exogenous heme using recombinant HO-expressing yeast cells was studied in this research. First, the heme oxygenase-1 gene (*HO1*) encoding the inducible plastidic isozyme from *Arabidopsis thaliana*, with the plastid transport peptide sequence removed, was recombined into *Pichia pastoris* GS115 cells. This resulted in the construction of a recombinant *P. pastoris* GS115-HO1 strain that expressed active HO1 in the cytoplasm. After that, the concentration of the inducer methanol, the induction culture time, the pH of the medium, and the concentration of sorbitol supplied in the medium were optimized, resulting in a significant improvement in the yield of HO1. Subsequently, the whole cells of GS115-HO1 were employed as catalysts to convert heme chloride (hemin) into biliverdin. The results showed that the yield of biliverdin was 132 mg/L when hemin was added to the culture of GS115-HO1 and incubated for 4 h at 30 °C. The findings of this study have laid a good foundation for future applications of this method for the economical production of biliverdin.

**Graphical Abstract:**

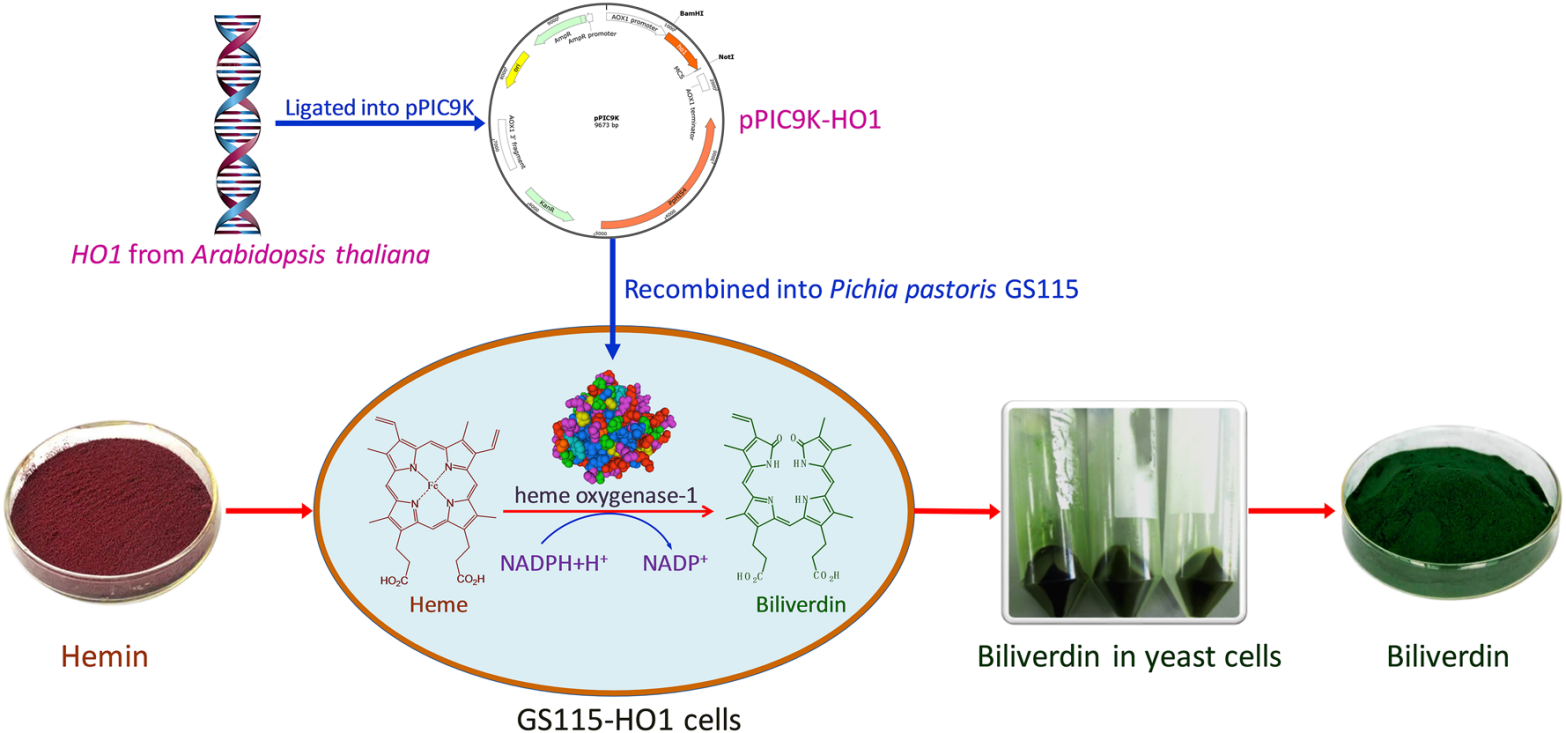

**Supplementary Information:**

The online version contains supplementary material available at 10.1186/s40643-024-00736-w.

## Introduction

**Fig. 1:**
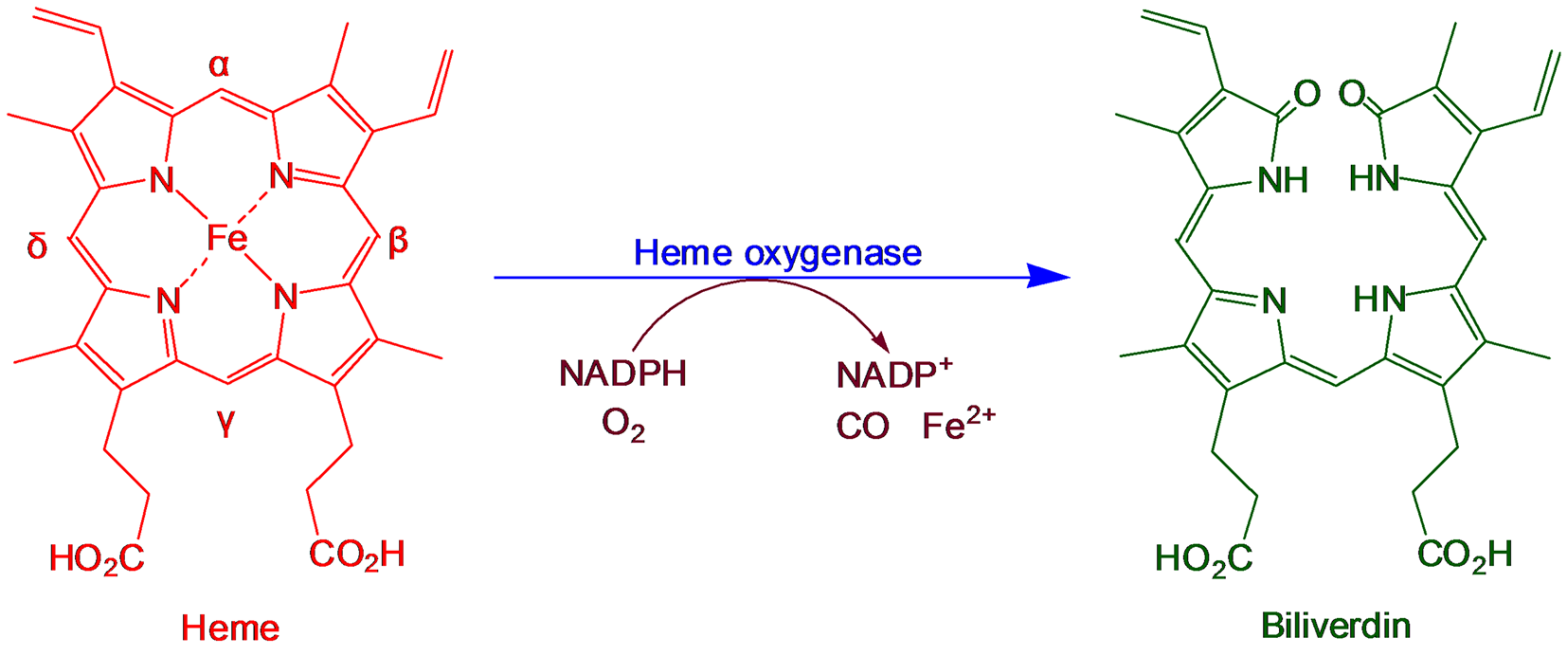
Pathway of heme metabolism into biliverdin by heme oxygenase

Biliverdin is one of the bile pigments that exist in many organisms. It is mainly produced through the degradation of heme. In the bodies of vertebrates, dead red blood cells release hemoglobin, which is then catabolized into globulin and heme. A selective cleavage of heme by heme oxygenase (HO; EC 1.14.99.3) occurs at the α-methane bridge, producing the biliverdin IXα isomer (Fig. 1). The term “biliverdin” typically refers to biliverdin IXα (McDonagh 2001). Biliverdin is subsequently reduced to bilirubin IXα by biliverdin reductase (EC 1.3.1.24). Three types of HO have been identified: oxygen stress-induced HO1, constitutive HO2, and an unidentified type HO3. Among them, HO1 has the highest catalytic activity towards heme and belongs to the heat shock protein family. Many microorganisms, plants, animals, and human cells contain HO1 (Celis and DuBois 2019).

As an intermediate metabolite in heme metabolism, biliverdin has long been regarded as a mere waste product excreted in bile. However, recent studies have revealed that it has many therapeutic effects on the human body, including antioxidant and anti-inflammatory properties (Vasavda et al. 2019; Shiels et al. 2020; McDonagh 2010; Gonzalez-Sanchez et al. 2016), suppression of the immune response (Baylor and Butler 2019; Homsher et al. 2018), stabilization of vascular endothelial cells (Nakao et al. 2005; Li et al. 2021), and regulation of cell apoptosis (Zheng et al. 2014; Moraes et al. 2012). Biliverdin is expected to be a potential therapeutic drug for many diseases. Additionally, biliverdin can also serve as a chromophore for certain materials (Uda et al. 2017; Fernandez-Rodriguez et al. 2017; Watermann et al. 2014), making it a significant application in the fields of materials science and synthetic biology.

Biliverdin naturally exists in bird eggshells and bile (Morales 2020; Halepas et al. 2017; Zhao et al. 2006), as well as in fish blood (Fang and Bada 1990) and bile (Ding and Xu 2002). However, the biliverdin content in these materials is low, and the cost of extracting it is high. Therefore, currently, reaction grade biliverdin is prepared by oxidation of bilirubin, but, because bilirubin is extracted from hog bile, the price is also high. Additionally, the many reaction steps and low yield of the oxidation process contribute to the high cost of biliverdin prepared using this method. Therefore, it is urgent to develop an economical and efficient production method for producing high-grade biliverdin. Recent developments in biosynthesis technology have brought new insights into the production of some natural products, and there has been considerable research in recent years to develop methods for the production of biliverdin (Chen et al. 2012; Pendrak and Roberts 2005; Yan et al. 2022; Seok et al. 2019).

When HO is expressed in *Escherichia coli* cells, the heme synthesized can be converted in situ into biliverdin (Ishikawa et al. 1991; Wilks and Ortiz de Montellano 1993). In Chen et al.'s (2012) study, *E. coli* expressing HO1 in batch fermentation achieved a biliverdin yield of 23.5 mg/L, without the need for an exogenous substrate. However, due to the limited ability of *E. coli* to synthesize heme, achieving a high yield of biliverdin by this route is challenging.

Heme is the immediate precursor of biliverdin and can be extracted in large quantities from animal blood at low cost. This makes producing biliverdin by converting heme through chemical or biological processes a promising strategy. Chemical hydrolysis has no stereoselectivity and yields four isomers of biliverdin (IXα, IXβ, IXγ, and IXδ). In contrast, biotransformation exhibits high selectivity and almost only produces biliverdin IXα through the enzymatic hydrolysis of heme by HO (McDonagh 2001). Several studies have reported the biotransformation of heme into biliverdin. Yan et al. (2022) constructed a HO1-expressing recombinant *E. coli*, which co-expressed glutamate dehydrogenase to achieve NADPH regeneration. The yield of biliverdin from heme was 71.5 mg/L. Using whole cells to convert heme into biliverdin, the main issue is that heme cannot enter the *E. coli* cells due to the absence of carrier proteins in its cell membranes (Naoe et al. 2017). Although Yan attempted to express HO1 by the surface display system, the improvement in biotransformation efficiency was unsatisfactory, with the yield of biliverdin increasing from 71.5 mg/L to only 76.3 mg/L.

Unlike *E. coli*, yeast cells can take up exogenous heme. For example, the addition of heme to the culture medium significantly enhanced the production of soybean leghemoglobin in recombinant *Pichia pastoris* (Chen et al. 2022). As early as 2005, Pendrak and Roberts (2005) applied for a patent in which they utilized a recombinant strain of the yeast *Candida albicans* expressing HO1 to produce biliverdin through the biotransformation of heme. Despite the fact that *C. albicans* cells have a strong ability to absorb heme, this yeast is an opportunistic human pathogen that presents certain challenges in industrial applications. Also, this patent does not provide a detailed description of the biotransformation method.

In the current paper, the HO1 gene (*HO1*) from the thale cress plant *Arabidopsis thaliana* was used to transform *P. pastoris*. This resulted in the expression of intracellular active HO1. The whole cells of recombinant *P. pastoris* were then utilized for the biotransformation of heme chloride (hemin) to produce biliverdin.

## Materials and methods

### Strains and plasmids

*Pichia pastoris* strain GS115 and plasmid pPIC9K were obtained from Yinyuan Biotech Co., Ltd (Hangzhou, China). *E. coli* strain DH5α was supplied by Beijing Tsingke Biotech Co., Ltd (Beijing, China).

### Reagents, enzymes and test kits

DNA restriction enzymes (BamHI, NotI, and SacI) were purchased from Takara Biomedical Technology Co., Ltd (Beijing, China). The Rapid Yeast Genomic DNA Isolation Kit, Plasmid DNA Mini-preps Kit, SDS-PAGE Preparation Kit, DNA Markers, Protein Markers, DNA Gel Extraction Kit, and the primers for α-factor, 5′-AOX, and 3′-AOX were supplied by Sangon Biotech Co., Ltd (Shanghai, China). TS GelRed Nucleic Acid Dye and T5 Super PCR Mix were obtained from Beijing Tsingke Biotech Co., Ltd (Beijing, China). Hemin (CAS: 16009-13-5, purity: 98%) and biliverdin hydrochloride (CAS: 55482-27-4, purity: 97%) were purchased from Aladdin Reagent Co., Ltd. (Shanghai, China). Geneticin (G418) was purchased from Invitrogen Corporation (Carlsbad, CA, USA). All other reagents were commercially available in analytical and biological grades.

### Culture media

Luria Broth medium: peptone 10 g/L, yeast extract 5 g/L, NaCl 10 g/L, pH 7.0. MD medium: glucose 20 g/L, yeast nitrogen base without amino acids (YNB) 13.4 g/L, biotin 0.4 mg/L, pH 6.0; YPD medium: peptone 20 g/L, yeast extract 10 g/L, glucose 20 g/L, pH 7.2. BMGY medium: peptone 20 g/L, yeast extract 10 g/L, glycerol 10 g/L, YNB 13.4 g/L, biotin 0.4 mg/L, prepared with pH 5.0, 0.1 mol/L sodium citrate buffer. BMMY medium: peptone 20 g/L, yeast extract 10 g/L, methanol 10 mL/L, YNB 13.4 g/L, biotin 0.4 mg/L, were adjusted to pH 5.0, with 0.1 mol/L sodium citrate buffer.

### Recombinant plasmid constructs

The DNA sequence encoding HO1 (Current name: TED4; Gene ID: 817208, sequence is shown in Additional file 1: Table S1) from *A. thaliana* was retrieved from the NCBI database (https://www.ncbi.nlm.nih.gov/), and the 159 bp upstream sequence expressing the plastid transit peptide, according to Shin's research (Shin et al. 2014), were removed. The protein sequence of the expressed HO1 is shown in Additional file 1: Table S2. The *HO1* gene was synthesized and ligated into the pPIC9K vector (between the BamHI and NotI restriction sites) by Beijing Qingke Biotech Co. Ltd. (Beijing, China), yielding the recombinant vector pPICZB-HO1, and it was propagated in *E. coli* strain DH5α.

### *P. pastoris* transformation and PCR analysis

Plasmid pPIC9K-HO1 was linearized using the SacI restriction enzyme. The reaction mixture consisted of 20 μL of pPIC9K-HO1 and 80 μL of GS115 competent cells, which were mixed and transferred into a pre-cooled electroporation cuvette (0.2-cm type). After being placed on ice for 5 min, the cells were electroporated for 4 ms in the “Fungi Pichia” mode using an electroporation device (MicroPulser 1,652,100, Bio-Rad, Philadelphia, PA, USA). After electroporation, 1 mL of pre-cooled 1 mol/L sorbitol was immediately added to the cuvette, and the cells were transferred to a 1.5-mL centrifuge tube, incubated in a water bath for 2 h at 30 °C, then plated onto MD agar plates containing 50 mg/L geneticin (G418), and cultured for 48 h at 30 °C.

Single colonies on the MD agar plates were picked and inoculated individually into 5 mL of YPD liquid medium containing 50 mg/L of G418. The cultures were incubated at 30 °C for 24 h, and then the total genomic DNA was extracted using the Rapid Yeast Genomic DNA Isolation Kit. The target genes were amplified by PCR using 5′-AOX and 3′-AOX as primers (Primer sequences are shown in Additional file 1: Table S1) and then sequenced for verification.

### Expression of HO1 and SDS-PAGE

The recombinant GS115-HO1 cell suspension (1 mL), stored in a 20% glycerol solution at − 80 °C, was inoculated into 50 mL of BMGY medium. It was then shake-cultured for 16 h at 30 °C and 180 r/min until the OD_600_ reached approximately 6.0. The culture was then transferred into a 100 mL sterile centrifuge tube and centrifuged at 5000×*g* for 5 min. The supernatant was discarded, and 25 mL of BMMY medium was added to re-suspend the cell pellet. The suspension was then transferred into a 250-mL sterile conical flask and shake-cultured for 3–5 days at 30 °C and 180 r/min to obtain the culture with an OD_600_ of 10–12. During culture, pure methanol was added as an inducer of *HO1* gene expression every 24 h to a final concentration of 1% (v/v) in the BMMY medium.

An aliquot of the GS115-HO1 culture (100 μL) was centrifuged at 8000×*g* for 5 min. The supernatant was then discarded, and the yeast cells were suspended in 50 μL deionized water and lysed using the “boiling-freezing-boiling” method. An aliquot (16 μL) of the lysate and 4 μL of 5 × protein loading buffer were mixed, incubated in boiling water for 10 min, and analyzed by SDS-PAGE using the SDS-PAGE Preparation Kit.

### HO1 activity determination

GS115-HO1 culture (5 mL) was centrifuged at 8000×*g* for 5 min, the supernatant was discarded, and 1 mL of pH 6.0, 0.1 mol/L sodium citrate buffer was added to resuspend the cells. The yeast cells were ultrasonically broken and then centrifuged at 8000×*g* at 4 °C for 10 min. The supernatant was collected, and the activity of the HO1 enzyme was determined.

The assay for measuring HO1 activity was as follows: 50 μL of 0.5 mmol/L hemin solution (dissolved in 0.05 mol/L Na_2_CO_3_), 449 μL of 0.1 mol/L pH 7.0 sodium citrate buffer, 40 μL of 10 g/L bovine serum albumen (BSA), 50 μL of 40 mmol/L d-glucose-6-phosphate disodium, 1 μL of 1 U/μL glucose-6-phosphate dehydrogenase solution, 10 μL of 20 mmol/L NADPH, 200 μL of the crude biliverdin reductase preparation, which was prepared according to the method described in the reference (Mei et al. 2022), and 200 μL of GS115-HO1 cell lysate were mixed in a 1.5-mL centrifuge tube and incubated for 15 min at 30 °C. The A_450_ of the enzyme reaction mixture was measured, and the concentration of bilirubin in it was calculated using the standard curve for bilirubin concentration—A_450_. The activity of the HO1 enzyme was then calculated as the amount of enzyme (in mL) required to produce 1 μg of bilirubin per minute at a temperature of 30 °C and pH of 7.0.

### Analysis of biliverdin

After the biotransformation, 25 mL of the reaction mixture was centrifuged at 8000×*g* for 10 min to separate the cells and liquid. The pelleted cells from the reaction mixture were suspended in 3 mL of methanol, shaken for 5 min, and centrifuged at 8000×*g* for 10 min. The supernatant was analyzed by high-performance liquid chromatography (HPLC) after being filtered through a 0.22-μm pore size membrane. The pH of the liquid from the reaction mixture was adjusted to 1.0 with 6 mol/L HCl, followed by centrifugation at 8000×*g* for 10 min. The supernatant was discarded and the precipitate dissolved in 3 mL of methanol for HPLC analysis after being filtered through a 0.22-μm pore size membrane.

Quantification of biliverdin was performed by HPLC using an Agilent Zorbax SB-C18 column (5 µm particle size, 150 mm × 4.6 mm, Agilent Technologies, Santa Clara, CA, USA) on a Shimadzu LC-20A System (Shimadzu, Kyoto, Japan). The mobile phase consisted of water containing 0.1% acetic acid (phase A) and methanol (phase B) with a gradient elution: 0–20 min, 40% A + 60% B to 100% B, and finally 20–30 min, 100% B, at a flow rate of 1 mL/min. The column temperature was 30 °C. The detection wavelength was 650 nm.

The biotransformation product was confirmed as biliverdin by liquid chromatography-electrospray ionization-mass spectrometry (LC–ESI–MS) on an LCQ Deca XP Plus mass spectrometer (Thermo-Fisher, Waltham, MA, USA). The scanning range was *m/z* 530–650, the capillary temperature was 300 °C, and the capillary voltage was 4.0 kV.

## Results and discussion

### Identification of the recombinant GS115-HO1

**Fig. 2:**
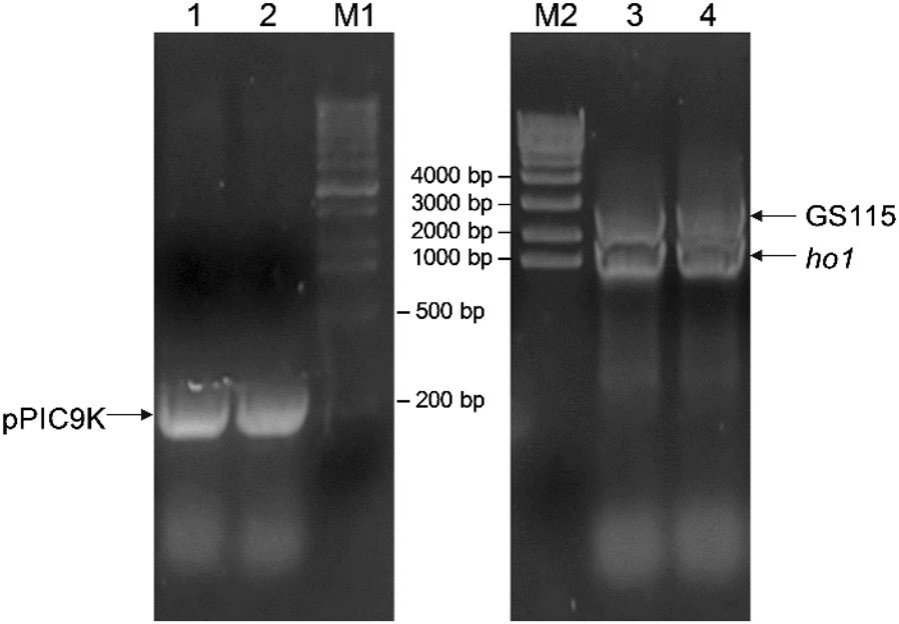
Confirmation of the presence of the *HO1* gene in the recombinant *Pichia pastoris* by PCR. Lanes 1 and 2: PCR products from the GS115-pPIC9K genome; lanes 3 and 4: PCR products from the GS115-HO1 genome; lanes M1 and M2: DNA markers

The genomes from cell suspensions GS115-pPIC9K and GS115-HO1 cultured in YPD liquid medium supplied with 50 mg/L G418 were separately extracted as PCR templates. The α-factor and 3′-AOX primers were used for PCR amplification of GS115-pPIC9K, whereas the 5′-AOX and 3′-AOX primers were used for PCR amplification of GS115-HO1. The results of agarose gel electrophoresis for the detection of PCR products are shown in Fig. 2.

In Fig. 2, the bands of the pPIC9K PCR product in lanes 1 and 2 were approximately 200 bp, which is consistent with the expected size of this plasmid (198 bp). In lanes 3 and 4, bands larger than 2000 bp represented the PCR products of the *P. pastoris* GS115 genome (2206 bp). The band of a size of approximately 1000 bp corresponded to the PCR product of the recombinant plasmid pPIC9K-HO1 (895 bp). The DNA in this band was recovered, and the sequencing results were compared and analyzed using BLAST. The results showed that this sequence was completely consistent with that of the target gene, indicating that *HO1* has been successfully recombined into GS115 cells.

### Expression of biliverdin reductase in recombinant *P. pastoris*

**Fig. 3:**
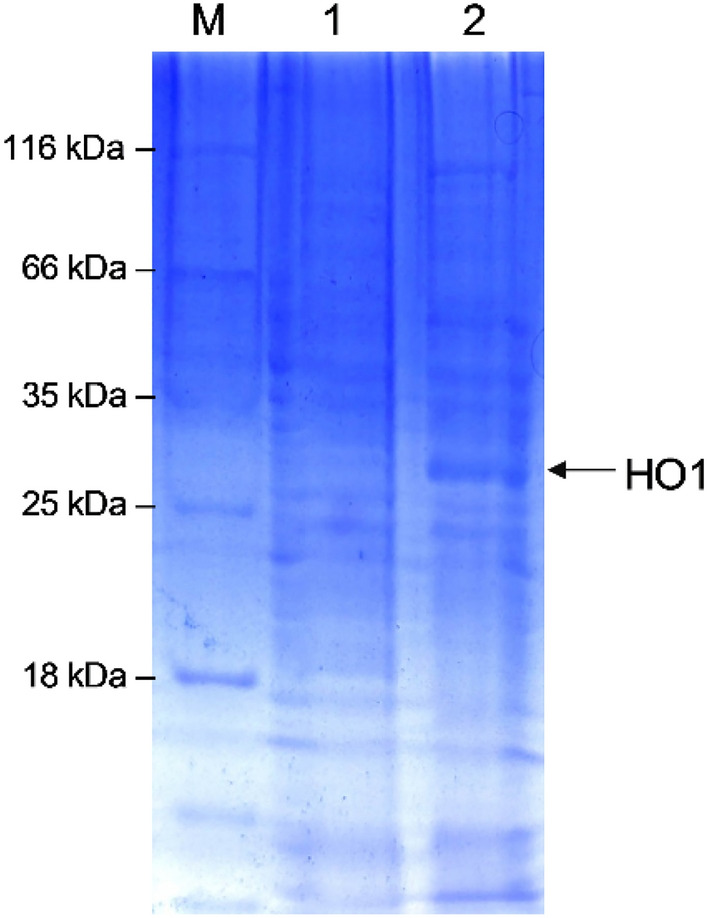
SDS-PAGE analysis of HO1 heterologously expressed by GS115-HO1. M: Protein markers; Lane 1: cell lysates of GS115-pPIC9K; lane 2: cell lysates of GS115-HO1

After culturing under conditions of methanol induction, the cells of GS115-pPIC9K and GS115-HO1 were disrupted using ultrasonication. The lysed cell suspension was then analyzed using SDS-PAGE. The electrophoretogram is shown in Fig. 3.

A protein with a molecular weight of approximately 27 kDa was detected in the lysates of GS115-HO1 cells (Fig. 3). The molecular weight of this protein was consistent with the expected molecular weight of HO1 (26.82 kDa, http://web.expasy.org/compute_pI/). In comparison, no such protein appeared in the lysates of GS115-pPIC9K cells. This indicates that GS115-HO1 is capable of expressing the recombinant protein HO1.The enzyme activity in the culture of the GS115-HO1 was 0.437 U/mL.

### Biotransformation of heme by recombinant *P. pastoris*

**Fig. 4:**
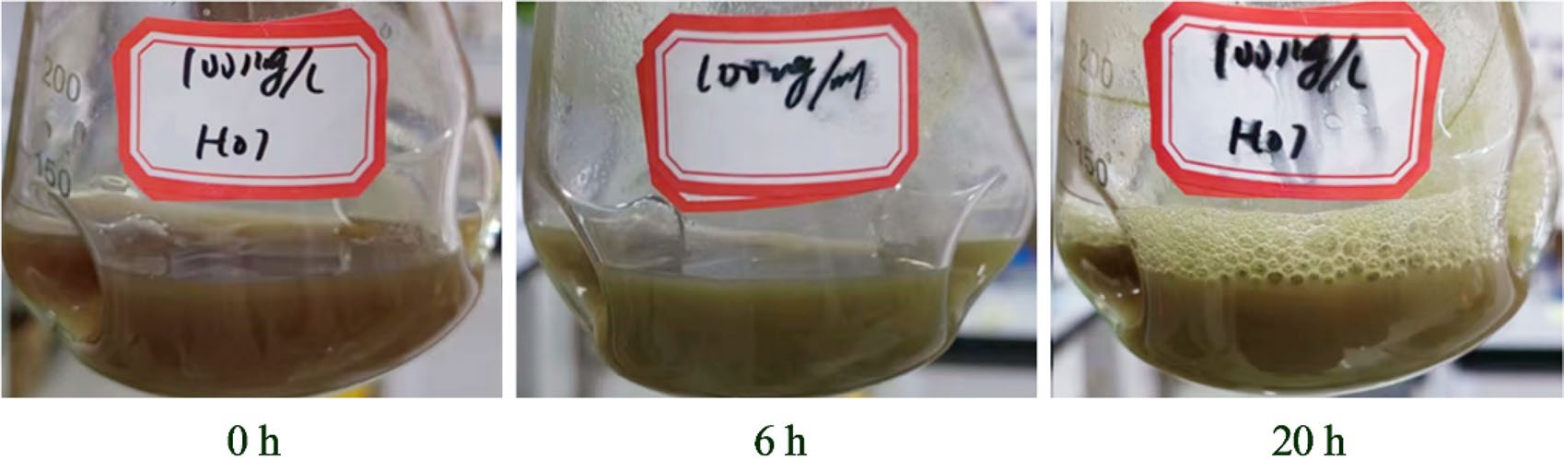
Images of GS115-HO1 culture after biotransformation for 0, 6, and 20 h in the presence of 0.1 g/L hemin

An aliquot (1 mL) of hemin solution, dissolved in 0.05 mol/L Na_2_CO_3_ at a concentration of 2.5 g/L, was added to a 24 mL culture of GS115-HO1, resulting in a hemin concentration of 0.1 g/L in the reaction mixture. The biotransformation was performed in a shaking incubator at 30 °C and 180 r/min. During the biotransformation process, it was observed that the color of the mixture gradually changed from brown to dark green. These color changes reflect that GS115-HO1 can convert hemin into biliverdin. However, if the biotransformation continued for more than 12 h, it further changes gradually to brownish-green, as biliverdin decomposed into other compounds. The images of the reaction mixture after biotransformation for 0, 6, and 20 h are shown in Fig. 4.

**Fig. 5:**
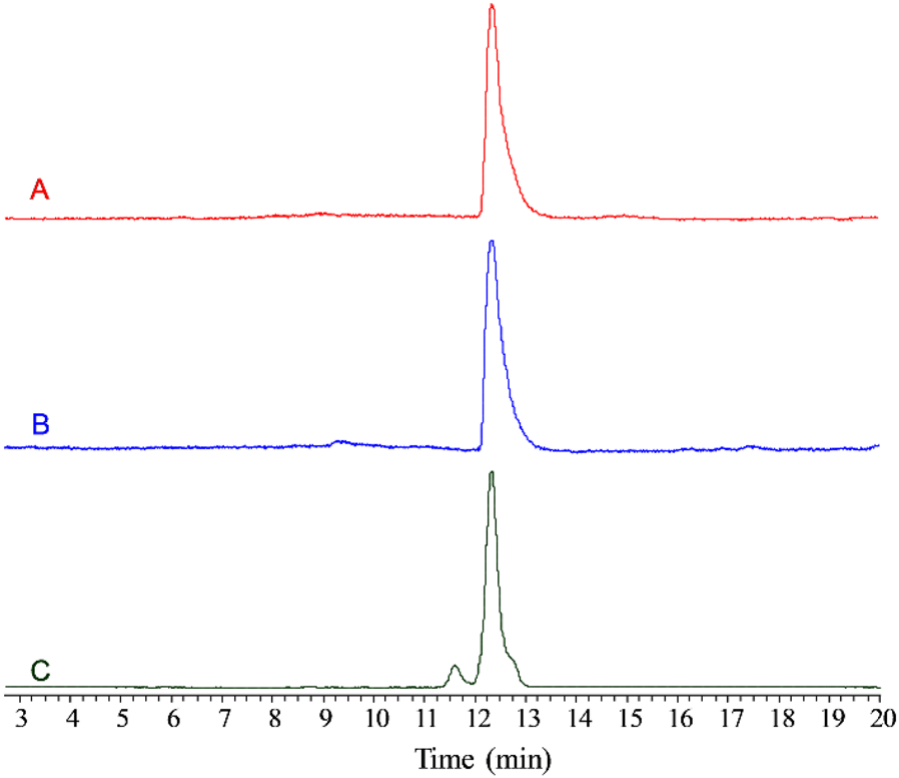
HPLC chromatograms of the products obtained from the biotransformation of hemin. A: Extracts of GS115-HO1 cells. B: Extracts from the supernatant of the GS115-HO1 culture; C: Biliverdin standard

**Fig. 6:**
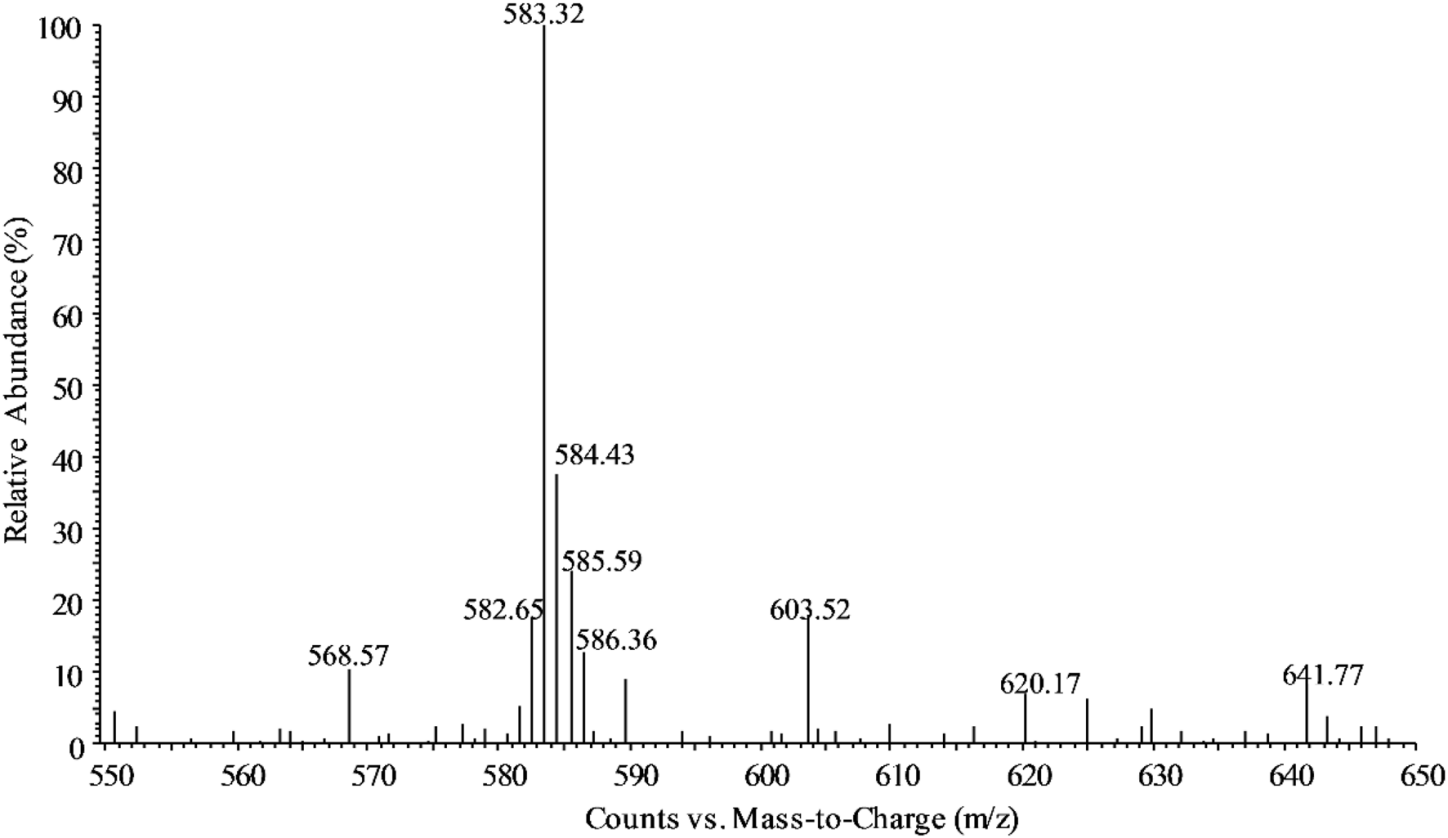
LC–ESI–MS spectra of the biotransformed biliverdin

The biotransformation product was analyzed using HPLC, and the chromatograms are shown in Fig. 5. It can be observed that the retention time of the green substances in both cells and supernatants is consistent with that of the biliverdin standards. LC–ESI–MS revealed a peak corresponding to [M + H]^+^ at *m/z* 583.32 (Fig. 6), indicating that the molecular weight of this compound is 582.32 Da, confirming that the biotransformation product is biliverdin.

### Optimization of HO1 expression conditions

**Fig. 7:**
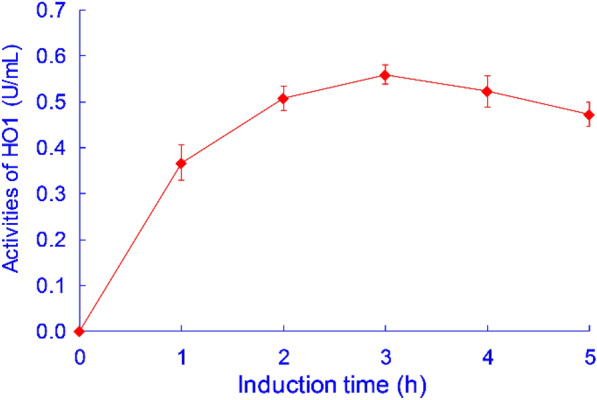
The activities of HO1 from GS115-HO1 under different induction times. The results were presented as the mean ± standard deviation (n = 3)

To improve the productivity of HO1 and achieve maximum activity, the pH of the BMMY medium (4.5, 5.0, 5.5, 6.0, 6.5, 7.0), the concentration of the inducer (0.5%, 1.0%, 1.5%, 2.0%, 2.5%, 3.0%, volume percentage of BMMY medium), the addition of sorbitol (0, 1.25%, 2.5%, 5.0%, 10%, molar ratio to methanol), and the duration of the induction period (1, 2, 3, 4, 5 d) were successively optimized in shake-flask experiments. The optimal expression conditions were obtained as follows: BMMY medium with a pH of 6.0, addition of 1% methanol, and a 2.5% molar ratio of sorbitol to methanol every 24 h during the induction culture. The induction process was conducted for 3 d, resulting in an enzyme activity of 0.560 U/mL in the GS115-HO1 culture. Compared to the enzyme activity of 0.437 U/mL before optimization, there was a 28.1% increase in activity. The activities of HO1 from GS115-HO1 under different induction times are shown in Fig. 7.

### Optimization of the conditions for the biotransformation of hemin into biliverdin

**Fig. 8:**
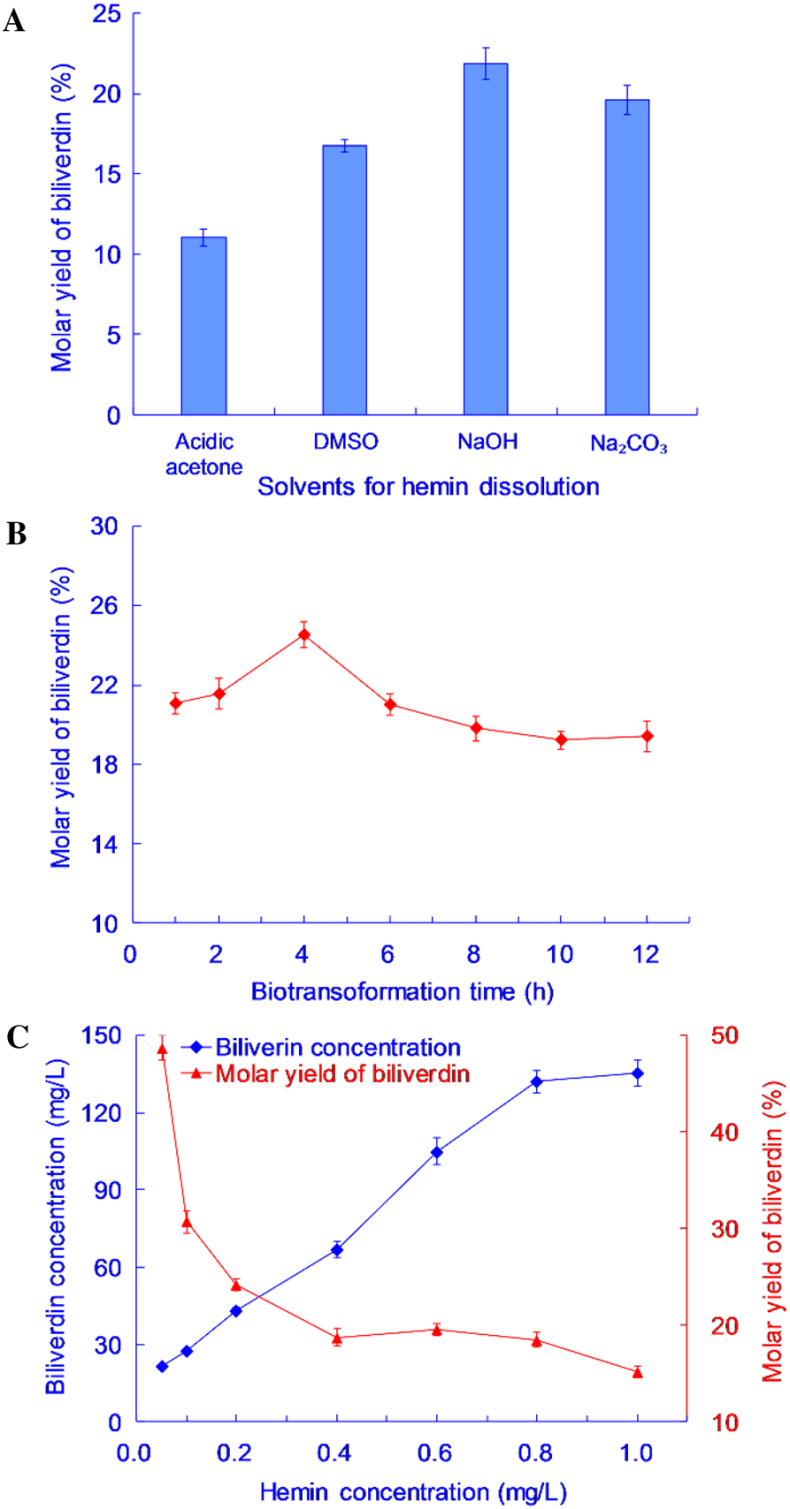
Optimization of the conditions for the biotransformation of hemin into biliverdin. **A**: Molar yields of biliverdin after biotransformation from hemin dissolved in different solvents. **B**: Time profile of biliverdin production through the biotransformation of hemin using whole cells of GS115-HO1. **C**: Production of biliverdin through the biotransformation of hemin by the whole cells of GS115-HO1 at different substrate concentrations. The results are presented as the mean ± standard deviation (n = 3)

Hemin is readily soluble in alkaline aqueous solutions, acidic acetone, and dimethyl sulfoxide (DMSO). In “Biotransformation of heme by recombinant *P. pastoris*” section, a Na_2_CO_3_ aqueous solution was used as the solvent for hemin. However, a significant amount of foam was generated under the shaking conditions of the biotransformation. The reason for the foaming is that CO_2_ was produced from Na_2_CO_3_ in an acidic BMMY medium, with the foam hindering the dissolution of oxygen in the culture medium. Therefore, 0.05 mol/L NaOH solution, DMSO, or acidic acetone (containing 1% volume of HCl) were investigated as solvents for dissolving hemin. An aliquot (1 mL) of hemin solution (5 g/L) was added to a 24 mL aliquot of GS115-HO1 culture, resulting in a final hemin concentration of 0.2 g/L in the reaction mixture. After 12 h of biotransformation at 30 °C and 180 r/min, the concentration of biliverdin in the reaction mixture was analyzed. The conversion yields of biliverdin in the different solvents are shown in Fig. 8A.

The conversion yield of biliverdin was highest when 0.05 mol/L NaOH aqueous solution was used as the solvent for adding hemin to the culture, compared with other solvents (Fig. 8A); when acidic acetone or DMSO were used as solvents for hemin, the conversion yields were lower than those achieved with NaOH or Na_2_CO_3_. This suggests that a high concentration (4%) of organic solvent is toxic to yeast cells. Therefore, the studies indicated that the most appropriate solvent for biliverdin was 0.05 mol/L NaOH solution.

In “Biotransformation of heme by recombinant *P. pastoris*” section, it had been reported that, if the biotransformation continued for more than 6 h, biliverdin decomposes into other compounds due to its instability under the ambient conditions of light and oxygen. Therefore, the time profile of biliverdin production needed to be determined. The biotransformation was performed with a hemin concentration of 0.2 g/L, temperature of 30 °C, and shaking speed of 180 r/min. The conversion yield of biliverdin is shown in Fig. 8B.

It can be seen that the conversion yield of biliverdin reached its peak after 4 h of biotransformation and then gradually decreased (Fig. 8B). Biliverdin is unstable under oxygenic conditions. The biotransformation was carried out under shaking conditions, and the concentration of dissolved oxygen in the reaction mixture was thus maintained at a high level, which may promote the degradation of biliverdin. Therefore, the optimal biotransformation time was 4 h, and the conversion yield of biliverdin over this period was 24.6%.

Aliquots of hemin solution in 0.05 mol/L NaOH at different hemin concentrations were added to a 24 mL GS115-HO1 culture, resulting in final hemin concentrations in each reaction mixture of 0.05, 0.1, 0.2, 0.4, 0.6, 0.8, or 1.0 g/L. The biotransformation was conducted in shaken batch cultures for 4 h at 30 °C and 180 r/min. The conversion yield of biliverdin is shown in Fig. 8C.

It was observed that, even when the substrate hemin concentration was as low as 0.05 g/L, the conversion yield of biliverdin was only 48.7%, and the concentration of biliverdin in the mixture was 21.9 mg/L (Fig. 8C). The concentration of biliverdin in the mixture increased with increasing hemin concentration, but the conversion yield decreased rapidly. When the concentration of hemin reached 0.8 g/L or above, the concentration of biliverdin remained almost constant at 132 mg/L, resulting in a conversion yield of 18.5% at 0.8 g/L hemin.

## Conclusion

In this study, the plastid transit peptide sequence of the *HO1* gene from *A. thaliana* was removed and the shortened coding sequence recombined into *P. pastoris* GS115. This resulted in the enzyme being expressed in the cytoplasm of the yeast cell but retaining its ability to convert exogenous heme (in the form of hemin) into biliverdin. The cells of the recombinant GS115-HO1 *P. pastoris* were used to achieve the biotransformation of hemin into biliverdin. Following optimization studies, the yield of biliverdin was 132 mg/L when hemin was added to the GS115-HO1 culture, which is the highest level reported in the literature to date. Although the yield of biliverdin was high, the conversion yield was only an unsatisfactory maximum of 18.5%, achieved at a hemin concentration of 0.8 g/L. The low conversion yield results in wasted substrate. However, considering that the price of biliverdin is more than ten times that of hemin in the current market, the low conversion yield is likely to be commercially acceptable. Additionally, this study did not test biotransformation in fermenters. With the use of fermenters, with sufficient oxygen and a continuous supply of methanol, the biomass of yeast cells will increase significantly. This may result in increased amounts of HO1 and a greater generation of NADPH, and the conversion yield of biliverdin would be expected to be improved.

### Supplementary Information

**Additional file 1: Table S1.** DNA sequences used in this study. **Table S2.** The protein sequence of the expressed HO1.

## Data Availability

All data generated or analyzed during this study are included in this article.
