# Supplementary material for: Production of biliverdin by biotransformation of exogenous heme using recombinant Pichia pastoris cells
Source: Bioresour Bioprocess. 2024 Feb 1;11(1):19. doi: 10.1186/s40643-024-00736-w (PMC10992137; doi:10.1186/s40643-024-00736-w)
Supplement: Supplementary file 1 — Additional file 1: Table S1. DNA sequences used in this study. Table S2. The protein sequence of the expressed HO1. [file 40643_2024_736_MOESM1_ESM.pdf]

Additional File 1

Production of biliverdin by biotransformation of exogenous heme using recombinant *Pichia pastoris* cells

Jianfeng Mei, Yanchao Han, Shihang Zhuang, Zhikai Yang, Yu Yi and Guoqing Ying*

College of Pharmaceutical Science, Zhejiang University of Technology, Hangzhou 310014, Zhejiang, China

* Corresponding author: Guoqing Ying

ORCID: 0000-0001-5683-7561

E-mail: bioph@zjut.edu.cn

Phone and fax: +86-571-88813460

Address: 18 Chaowang Road, Gongshu District, Hangzhou 310014, China

Table S1. DNA sequences used in this study

Name	Sequence (5’ → 3’)
TED4 (Gene ID: 817208)	ATGGCGTATTTAGCTCCGATTTCTTCATCCTTATCCATATTCAAGAAT<br>CCCCAACTCTCAAGATTCCAATTTTCTTCTTCCTCACCGAACCCACT<br>TTTCCTTCGACCTAGGATTCAGATTCTGAGTATGACCATGAACAAGT<br>CGCCGTCTTTAGTGGTGGTTGCGGCTACTACTGCGGCAGAGAAGCA<br>GAAGAAGAGGTATCCTGGAGAATCAAAGGGTTTTGTGGAGGAGAT<br>GAGGTTTGTGGCTATGAGACTTCATACTAAAGATCAAGCTAAGGAA<br>GGTGAGAAAGAGACTAAATCTATTGAGGAACGTCCTGTTGCTAAAT<br>GGGAACCTACTGTTGAAGGTTACTTGAGGTTTCTTGTGGATAGTAA<br>ATTGGTTTATGATACTCTTGAACTGATTATTCAAGACTCCAATTTCCC<br>AACTTATGCCGAGTTCAAGAACACGGGGCTGGAAAGAGCGGAGAA<br>ATTATCCACGGATTTGGAGTGGTTCAAGGAACAAGGTTACGAGATT<br>CCAGAACCAACAGCTCCTGGTAAAACATATTCTCAATATTTAAAGG<br>AATTAGCAGAGAAGGATCCTCAAGCATTCATTTGTCACTTCTACAA<br>CATCTACTTTGCTCATAGTGCTGGTGGACGAATGATTGGCAGAAAG<br>GTGGCAGAGCGGATACTCGATAATAAAGAACTCGAGTTCTACAAAT<br>GGGACGGCGAACTTTCTCAATTGTTGCAGAACGTTAGGGAGAAAC<br>TGAACAAGGTTGCAGAGGAGTGGACTAGAGAAGAAAAGAATCATT<br>GTTTGGAAGAGACTGAGAAATCGTTCAAGTATTCTGGTGAGATACT<br>TCGTCTCATATTGTCCTGA
Truncated sequences of *TED4*	ATGGCGGCTACTACTGCGGCAGAGAAGCAGAAGAAGAGGTATCCT<br>GGAGAATCAAAGGGTTTTGTGGAGGAGATGAGGTTTGTGGCTATG<br>AGACTTCATACTAAAGATCAAGCTAAGGAAGGTGAGAAAGAGACT<br>AAATCTATTGAGGAACGTCCTGTTGCTAAATGGGAACCTACTGTTG<br>AAGGTTACTTGAGGTTTCTTGTGGATAGTAAATTGGTTTATGATACT<br>CTTGAACTGATTATTCAAGACTCCAATTTCCCAACTTATGCCGAGTT<br>CAAGAACACGGGGCTGGAAAGAGCGGAGAAATTATCCACGGATTT<br>GGAGTGGTTCAAGGAACAAGGTTACGAGATTCCAGAACCAACAGC<br>TCCTGGTAAAACATATTCTCAATATTTAAAGGAATTAGCAGAGAAG

	GATCCTCAAGCATTCATTTGTCACTTCTACAACATCTACTTTGCTCAT AGTGCTGGTGGACGAATGATTGGCAGAAAGGTGGCAGAGCGGATA CTCGATAATAAAGAACTCGAGTTCTACAAATGGGACGGCGAACTTT CTCAATTGTTGCAGAACGTTAGGGAGAAACTGAACAAGGTTGCAG AGGAGTGGACTAGAGAAGAAAAGAATCATTGTTTGGAAGAGACTG AGAAATCGTTCAAGTATTCTGGTGAGATACTTCGTCTCATATTGTCC TGA
5'-AOX primer	GACTGGTTCCAATTGACAAGC
3'-AOX primer	GCAAATGGCATTCTGACATCC
α-factor primer	TACTATTGCCAGCATTGCTGC

Table S2. The protein sequence of the expressed HO1

Name	Protein sequence
Truncated heme oxygenase-1	MAATTAAEKQKKRYPGESKGFVEEMRFVAMRLHTKDQAKEGEKETKSIEE RPVAKWEPTVEGYLRFLVDSKLVYDTLELIIQDSNFPTYAEFKNTGLERAEK LSTDLEWFKEQGYEIPEPTAPGKTYSQYLKELAEKDPQAFICHFYNIYFAHS AGGRMIGRKVAERILDNKELEFYKWDGELSQLLQNVREKLNKVAEEWTR EEKNHCLEETEKSFKYSGEILRLILS
